# Invisible Hyperbolic Metamaterial Nanotube at Visible Frequency

**DOI:** 10.1038/srep16027

**Published:** 2015-11-02

**Authors:** Kyoung-Ho Kim, You-Shin No, Sehwan Chang, Jae-Hyuck Choi, Hong-Gyu Park

**Affiliations:** 1 Department of Physics, Korea University, Seoul 136-701, Republic of Korea

## Abstract

Subwavelength-scale metal and dielectric nanostructures have served as important building blocks for electromagnetic metamaterials, providing unprecedented opportunities for manipulating the optical response of the matter. Recently, hyperbolic metamaterials have been drawing particular interest because of their unusual optical properties and functionalities, such as negative refraction and hyperlensing of light. Here, as a promising application of a hyperbolic metamaterial at visible frequency, we propose an invisible nanotube that consists of metal and dielectric alternating thin layers. The theoretical study of the light scattering of the layered nanotube reveals that almost-zero scattering can be achieved at a specific wavelength when the transverse-electric- or transverse-magnetic-polarized light is incident to the nanotube. In addition, the layered nanotube can be described as a radial-anisotropic hyperbolic metamaterial nanotube. The low scattering occurs when the effective permittivity of the hyperbolic nanotube in the angular direction is near zero, and thus the invisibility of the layered nanotube can be efficiently obtained by analyzing the equivalent hyperbolic nanotube. Our new method to design and tune an invisible nanostructure represents a significant step toward the practical implementation of unique nanophotonic devices such as invisible photodetectors and low-scattering near-field optical microscopes.

The suppression of the visibility of an object is one of the most exciting optical phenomena. Recent advances in material sciences have accelerated the development of camouflages and invisible sensors by introducing plasmonic materials or electromagnetic metamaterials, which consist of subwavelength-sized metal and dielectric nanostructures^1^,^2^,^3^,^4^,^5^,^6^,^7^,^8^,^9^,^10^. Invisible optical sensors, such as gold-covered Si nanowires^11^, and various types of near-zero permittivity metamaterials^12^,^13^,^14^,^15^, have been proposed for enhancing the optical signal detection and simultaneously reducing the light scattering from the detectors. Plasmonic cloaking based on dipole field scattering cancellation occurring in deep subwavelength-sized core/shell nanoparticles has also been investigated^7^,^8^,^9^,^10^. In particular, near-zero permittivity has been experimentally demonstrated in one class of metamaterials, called hyperbolic metamaterials^16^,^17^,^18^. Hyperbolic metamaterials are of special interest because of their unusual properties, such as the hyperbolic nature of dispersion and strong optical anisotropy of permittivity^19^,^20^,^21^,^22^, which enable subwavelength imaging^23^,^24^,^25^,^26^, focusing^26^,^27^, photon lifetime engineering^28^, and spontaneous emission enhancement^29^. However, despite their intriguing potential, particularly for invisibility devices, the rational design rules of the hyperbolic metamaterials have not been widely explored yet.

In this study, we propose a wavelength-scale invisible nanotube at visible frequency that consists of alternating metal and dielectric thin layers. The designed metal and dielectric thin layers allow light propagation through the nanotube without distortion of the incident light. The theoretical study of the light scattering shows that almost zero scattering is observed in the layered nanotube at specific wavelengths of both transverse-electric (TE) and transverse-magnetic (TM) polarized incident light. In addition, to better understand such a low scattering, we modeled the layered nanotube as a radial-anisotropic hyperbolic metamaterial nanotube and examined the light scattering behavior. We found that the low scattering occurs when the effective permittivity of the hyperbolic nanotube in the angular direction is near zero, which is distinguishable from that of conventional plasmonic cloaking. Therefore, by analyzing the equivalent hyperbolic nanotube, the characteristic invisibility of the layered nanotube can be efficiently designed and tuned, and further used for practical implementation of unique nanophotonic devices.

## Results

### Invisible metal-dielectric layered single nanotube

**Figure 1:**
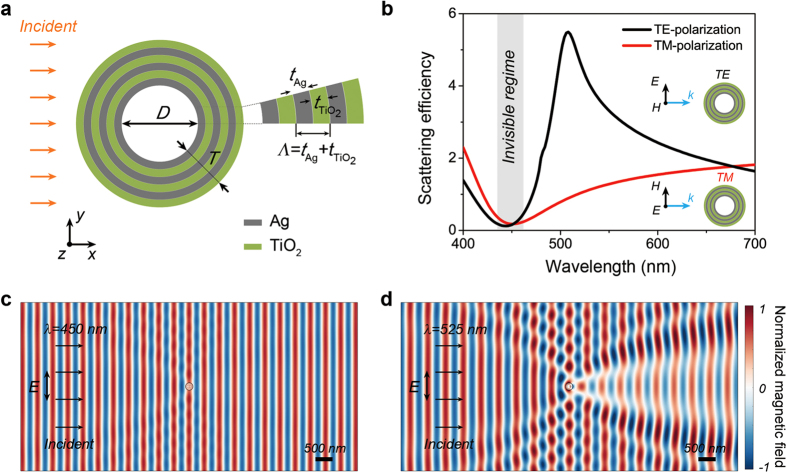
Light scattering in a metal-dielectric layered single nanotube. (**a**) Schematic illustration of a metal-dielectric layered nanotube with circular cross section. The shell is composed of alternating Ag (gray) and TiO_2_ (green) layers; *t*_Ag_, *t*_TiO2_, and *Λ* are the thicknesses of the Ag and TiO_2_ layers, and the period, respectively. (**b**) Scattering efficiency spectra of a layered nanotube with an air core diameter (*D*) of 100 nm, Ag layer thickness (*t*_Ag_) of 10 nm, TiO_2_ layer thickness (*t*_TiO2_) of 10 nm, and total shell thickness (*T*) of 60 nm. The filling ratio of the Ag layer (*f*_Ag_) is 0.5. Transverse-electric (TE, black) and transverse-magnetic (TM, red) polarized plane waves are normally incident to the nanotube. The shaded region of the spectra indicates the low-scattering invisible regime for both polarization directions. The inset shows the field directions for TE- and TM-polarized incident light. (**c**) A time snapshot of the normalized magnetic field (*H*_*z*_) distribution surrounding the layered nanotube for the TE-polarized incident light with a wavelength of 450 nm. (**d**) The same *H*_z_ field distribution as that shown in (**c**), but for a wavelength of 525 nm. The scale bars are 500 nm.

We designed an infinitely long metal-dielectric layered nanotube with a circular cross section and investigated the light scattering properties by applying a normal incident plane wave (Fig. 1a). The nanotube has an air core region with a diameter *D* and a layered shell structure with total thickness *T*. The shell structure consists of multiple alternating layers of Ag and TiO_2_, whose thicknesses are *t*_Ag_ and *t*_TiO2_, respectively. The period of the alternating Ag/TiO_2_ layered structure, *Λ*, is defined as *t*_Ag_ + *t*_TiO2_, and the filling ratio of the Ag layer in a period, *f*_Ag_, is defined as *t*_Ag_/*Λ*. The material composition was chosen to efficiently control the optical properties of the structure, as the signs of the Ag and TiO_2_ permittivities are opposite at visible frequencies (see Supplementary Figure S1 and Methods for details). Furthermore, the optical properties of the alternating layered structure can be tuned by adjusting the thicknesses of the Ag and TiO_2_ layers.

We investigated the optical properties of the layered nanotube structure with a core diameter of 100 nm and a shell thickness of 60 nm, where the thicknesses of both the individual Ag and TiO_2_ layers are set to 10 nm. As shown in Fig. 1b, the scattering efficiency was calculated as a function of the wavelength for TE- or TM-polarized light normally incident to the side of the nanotube by using the analytical Lorentz-Mie light scattering method (Fig. 1b) (Methods)^30^,^31^. Interestingly, the calculated scattering efficiency spectra show a dramatic reduction at a wavelength of ~450 nm for both polarizations: the visibility of the nanotube structure is significantly lowered in this reduced scattering regime (gray region in Fig. 1b). To clearly observe such invisibility of the nanotube, we obtained the normalized magnetic field distribution surrounding the nanotube at the wavelength of 450 nm for the TE-polarized incident light, using the finite element full-wave simulation method (Fig. 1c) (Methods). For comparison, the normalized magnetic field distribution was also calculated at the different wavelength of 525 nm, showing the maximum scattering efficiency, for the same layered nanotube and with the same polarization direction of the incident light (Fig. 1d). At the incident wavelength of 450 nm, the interference pattern induced by the scattered light from the nanotube is negligible, which stems from the destructive interference of the scattered light waves caused by the metal and dielectric layers. For TM-polarized incident light, there is also the destructive interference of the scattering from the different layers. Consequently, the entire field distribution is not distorted, which can explain the significant suppression of the scattering signal from the layered nanotube. On the other hand, at the incident wavelength of 525 nm, the constructive interference of the scattered waves from the metal and dielectric layers occurs, and thus, field distortion and strong light scattering by the nanotube can be observed.

**Figure 2:**
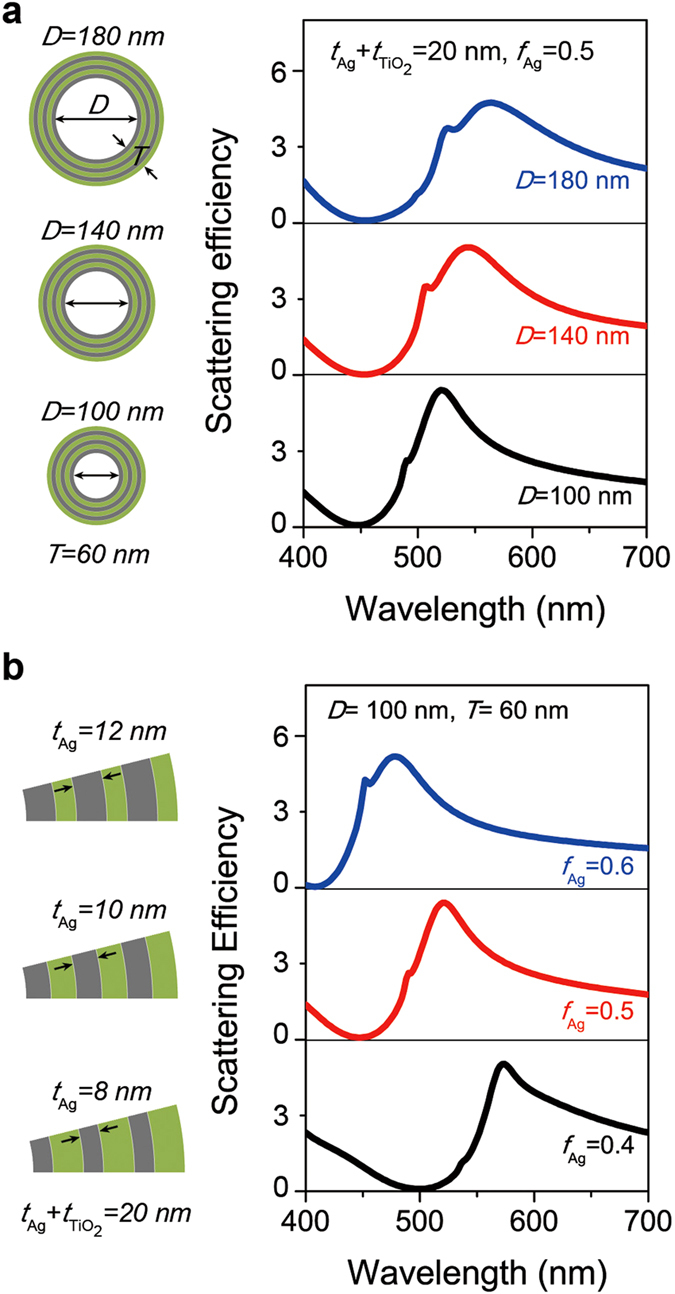
Scattering characteristics of the layered nanotubes with different core diameters and filling ratios. (**a**) Scattering efficiency spectra from layered nanotubes with different air core diameters (*D*) for transverse-electric (TE) polarized incident light. *D* is changed from 100 nm (bottom) to 180 nm (top) while the total shell thickness (*T*), Ag layer thickness (*t*_Ag_), and TiO_2_ layer thickness (*t*_TiO2_) are fixed to 60, 10, and 10 nm, respectively. (**b**) Scattering efficiency spectra from the layered nanotubes with different Ag filling ratios (*f*_Ag_) for TE-polarized incident light. *f*_Ag_ is changed from 0.4 (bottom, *t*_Ag_ = 8 nm) to 0.6 (top, *t*_Ag_ = 12 nm) while *D, T*, and the period (*Λ*) are fixed to 100, 60, and 20 nm, respectively.

To further characterize the optical properties and scattering behavior of the layered nanotube, we systematically changed the structural parameters of the layered nanotube and performed the same calculations as in Fig. 1b. Figure 2a shows the scattering efficiency spectra of the layered nanotubes with different air core diameters for TE-polarized incident light. The core diameter was varied from 100 to 180 nm while *T, t*_Ag_, and *t*_TiO2_ were fixed to 60, 10, and 10 nm, respectively. We note that the scattering efficiencies show similar spectral behaviors regardless of the variation of the air core diameters and the overall size of the nanotube, provided that the structural composition of the layered shell remains unchanged. The absolute values of the scattering efficiency at each wavelength are also all similar. In particular, the fixed invisible regime at the wavelength of ~450 nm was clearly observed for all the different core diameters, if the overall size of the nanotube is smaller than the wavelength. Additionally, we calculated the scattering efficiencies of the same nanotube structures for TM-polarized incident light: no significant changes in light scattering for all core diameters were observed (Supplementary Figure S2a). Such robustness of invisibility for the variation of the core diameter is a remarkable characteristic of our layered nanotube, compared to previous results obtained with the scattering cancellation method, which imposes strict conditions on the ratio of the core to the outermost diameter^7^,^10^.

Figure 2b shows the calculated scattering efficiencies of the layered nanotubes with different Ag layer filling ratios for TE-polarized incident light. For a fixed *Λ* of 20 nm, we varied *f*_Ag_ from 0.4 to 0.6; consequently, *t*_Ag_ was changed from 8 to 12 nm and *t*_TiO2_ was changed from 12 to 8 nm, while the air core diameter (100 nm), the total shell thickness (60 nm), and the number of alternating Ag/TiO_2_ layers remained unchanged. In contrast to the air core diameter variation, the overall light scattering behavior changes dramatically as *f*_Ag_ varies. Especially, the strong scattering suppression in the invisible wavelength regime redshifts from ~410 to ~500 nm as *f*_Ag_ decreases from 0.6 to 0.4, which reveals the highly sensitive light scattering nature of our nanotube with respect to the small variation of a few nanometers in the layered shell structure. For TM-polarized incident light, the spectral position of the scattering suppression agrees well with that observed for TE-polarized light and is also accordingly redshifted as *f*_Ag_ decreases (Supplementary Figure S2b). Therefore, the unique invisibility characteristic of the metal-dielectric layered nanotube significantly depends on the material composition in the layered shell structure, rather than the air core diameter. Furthermore, this reveals that the destructive interference of the scattered waves from the metal and dielectric layers is more significantly affected by the variation of the composite ratio of each layer rather than the uniform change of the overall size of the nanotube. Therefore, one can efficiently tune the invisibility of the layered nanotube by controlling the ratio of the Ag layer thickness to the period of the alternating layered media.

### Radial-anisotropic hyperbolic metamaterial nanotube

**Figure 3:**
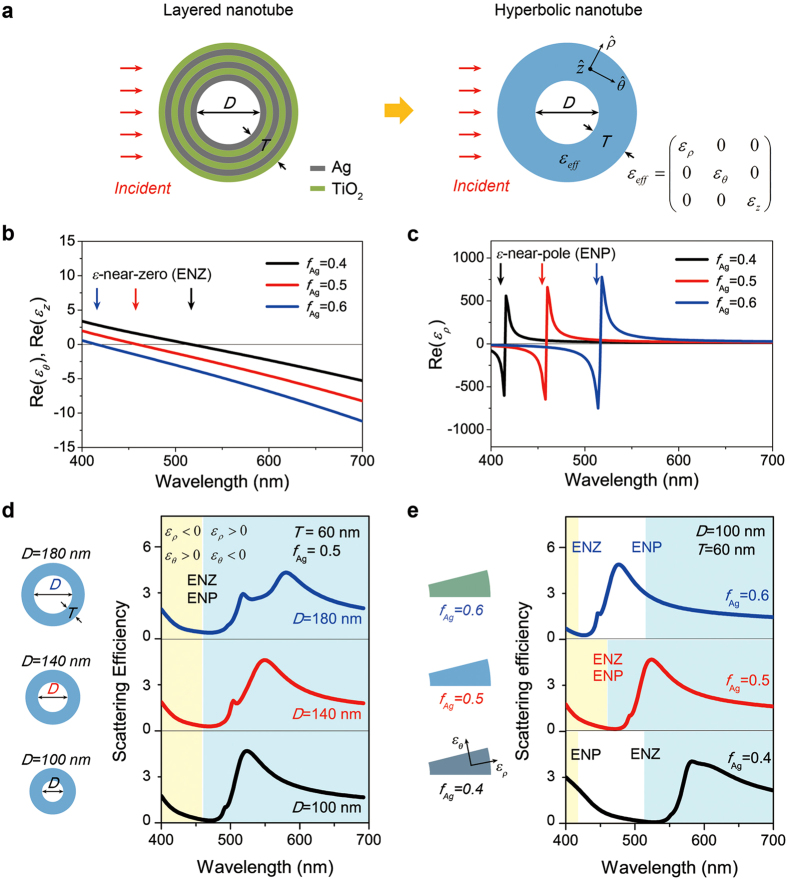
Scattering properties of a radial-anisotropic hyperbolic metamaterial nanotube. (**a**) Schematic diagram of the layered nanotube and corresponding radial-anisotropic hyperbolic metamaterial nanotube. (**b**) Real part of the effective permittivity in the *θ-* and *z-*directions (*ε*_*θ*_ and *ε*_*z*_) of the hyperbolic metamaterial nanotube with a variation of the filling ratio of the Ag layer (*f*_Ag_) from 0.4 to 0.6. The black, red and blue arrows indicate the *ε*-near-zero (ENZ) wavelengths at *f*_Ag_ of 0.4, 0.5, and 0.6, respectively. (**c**) Real part of the effective permittivity in the *ρ*-direction (*ε*_*ρ*_) of the hyperbolic metamaterial nanotube with a variation of *f*_Ag_ from 0.4 to 0.6. The black, red, and blue arrows indicate the *ε*-near-pole (ENP) wavelengths at *f*_Ag_ of 0.4, 0.5, and 0.6, respectively. (**d**) Scattering efficiency spectra of the hyperbolic metamaterial nanotubes with different core diameters for transverse-electric (TE) polarized incident light. The air core diameter (*D*) is equal to 100 nm (bottom), 140 nm (middle), and 180 nm (top), while the total shell thickness (*T*) and *f*_Ag_ are fixed to 60 nm and 0.5, respectively. *ε*_*ρ*_ is negative and *ε*_*θ*_ is positive in the yellow shaded region, whereas *ε*_*ρ*_ is positive and *ε*_*θ*_ is negative in the blue shaded region. (**e**) Scattering efficiency spectra of the hyperbolic metamaterial nanotube with different filling ratios, for TE-polarized incident light. *f*_Ag_ is equal to 0.4 (bottom), 0.5 (middle), and 0.6 (top), while *D* and *T* are fixed to 100 nm and 60 nm, respectively. The yellow and blue shaded regions also indicate the hyperbolic dispersion regimes.

To achieve a more fundamental understanding of the calculation results, which show that the characteristic invisibility of the metal-dielectric layered nanotube depends significantly on the material composition, we modeled the layered nanotube as a radial-anisotropic hyperbolic metamaterial nanotube with an effective permittivity tensor (Fig. 3a). In previous works, the optical properties of hyperbolic metamaterials with various two-dimensional or three-dimensional bulk structures were studied^21^,^22^,^32^,^33^. Additionally, metamaterials consisting of alternating metal-dielectric layers with cylindrical symmetry are known to possess an anisotropic permittivity in the radial direction^23^,^24^. In our model, the hyperbolic metamaterial nanotube is set to have the same air core diameter and total shell thickness as the alternating layered nanotube; however, the shell is composed of a single material with a diagonal effective permittivity tensor, *ε*_eff_. The principal components in the *ρ*-, *θ*-, and *z*-directions (*ε*_*ρ*_, *ε*_*θ*_, and *ε*_*z*_, respectively) of *ε*_eff_ are given by


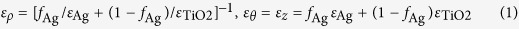


where *ε*_Ag_ and *ε*_TiO2_ are the permittivities of the Ag and TiO_2_ layers at a given frequency, respectively^22^,^34^. In Fig. 3b, we plotted the real parts of *ε*_*θ*_ and *ε*_*z*_ as functions of the wavelength in the visible frequency regime, as *f*_Ag_ varies from 0.4 to 0.6. We note that the two principal components of the *θ*- and *z*-directions in the effective permittivity tensor smoothly increase from negative to positive values as the wavelength decreases, which certainly shows one of the conventional characteristics of optical metamaterials^17^,^18^. Furthermore, the *ε*_*θ*_ and *ε*_*z*_ dispersion curves cross the point at which the effective permittivity values are zero, called *ε*-near-zero (ENZ). The arrows in Fig. 3b indicate each ENZ wavelength at a different *f*_Ag_: the ENZ wavelengths are shifted to shorter wavelengths as *f*_Ag_ increases. On the other hand, Fig. 3c shows the other principal component of the *ρ*-direction in the effective permittivity tensor plotted by using the values of *f*_Ag_ in Fig. 3b. In contrast to the monotonous changes in the *θ*- and *z*-directions, the *ε*_*ρ*_ curves show a singular dispersion behavior at certain frequencies where the sign of *ε*_*ρ*_ changes abruptly. The arrows in Fig. 3c indicate each singularity wavelength of the effective permittivity dispersion at a different *f*_Ag_, which is called *ε*-near-pole (ENP). The ENP wavelengths are shifted to longer wavelengths as *f*_Ag_ increases. From the results in Fig. 3b,c, one can easily find the spectral regimes where one of the principal components of the effective permittivity tensor is opposite in sign to the other two principal components, which directly indicates the formation of a hyperbolic dispersion and the radial-anisotropic nature of our modeled nanotube.

Next, we calculated the scattering efficiencies of our modeled hyperbolic metamaterial nanotube by using an analytical Lorentz-Mie scattering method^35^,^36^. We used the permittivity tensor obtained in Fig. 3b,c for the nanotube structure and applied TE-polarized normal incident light. Figure 3d,e show the scattering efficiencies of the hyperbolic metamaterial nanotube for different core diameters and Ag layer filling ratios, respectively. First, *D* was changed from 100 (bottom) to 180 nm (top), while *f*_Ag_ and *T* were fixed to 0.5 and 60 nm, respectively (Fig. 3d). Interestingly, for a fixed *f*_Ag_ of 0.5, the scattering efficiency spectra showed a significant scattering reduction at a wavelength of ~470 nm for all core diameters at which the ENZ and ENP are coincidently formed. Notably, not only the overall scattering behaviors, but also the characteristic invisibility of the hyperbolic metamaterial nanotubes in Fig. 3d qualitatively agrees with the results obtained for the metal-dielectric layered nanotubes in Fig. 2a. Although there are slight differences in the spectral positions of invisibility and other peaks between the hyperbolic and the layered nanotubes, these deviations originate from the effective medium approximation of the layered nanotube introduced by using Eq. (1). By employing thinner metal and dielectric layers, the wavelength difference will decrease (see Supplementary Figure S3 for details).

Second, the scattering properties of the hyperbolic metamaterial nanotube were re-examined when *f*_Ag_ was changed from 0.4 (bottom) to 0.6 (top), but *D* and *T* were fixed to 100 nm and 60 nm, respectively (Fig. 3e). As *f*_Ag_ varied, the scattering spectra changed remarkably. Interesting features are as follows. (1) A strong scattering suppression in each spectrum is achieved at the wavelengths of 530, 470, and 425 nm when the *f*_Ag_ values are 0.4, 0.5, and 0.6, respectively. Notably, the invisibility of the hyperbolic metamaterial nanotube occurs at the spectral position where the ENZ is formed. (2) The ENZ and ENP are not coincidently located in the same spectral position unless the *f*_Ag_ value is 0.5, as shown in Fig. 3b,d. Therefore, there are spectral ranges where the nanotube loses its hyperbolic nature when the *f*_Ag_ value is either 0.4 or 0.6. Here, the two hyperbolic regimes are denoted by yellow and blue shaded colors in Fig. 3d,e: *ε*_*ρ*_ is negative and *ε*_*θ*_ is positive in the yellow region, whereas the opposite signs are applied in the blue region. The unshaded region indicates the elliptical dispersion where both *ε*_*ρ*_ and *ε*_*θ*_ are either positive or negative. (3) More importantly, the overall spectral behavior and the absolute values of the invisible wavelengths are all in good agreement with those of the spectra in Fig. 2b. The slightly redshifted invisible wavelength of the hyperbolic metamaterial nanotube, compared to that of the layered nanotube, is due to the effective medium approximation. Thus, based on the observations and analyses in Figs 2 and 3, we conclude that the proposed model of hyperbolic metamaterial nanotube is highly useful for describing the characteristic scattering behaviors of a metal-dielectric layered nanotube. Furthermore, we found that the invisibility of the hyperbolic metamaterial nanotube occurs when *ε*_*θ*_ is nearly zero, which enables an efficient manipulation of the light scattering in the hyperbolic metamaterial nanotube by carefully tuning *ε*_*θ*_ to be near zero at any desired wavelengths.

**Figure 4:**
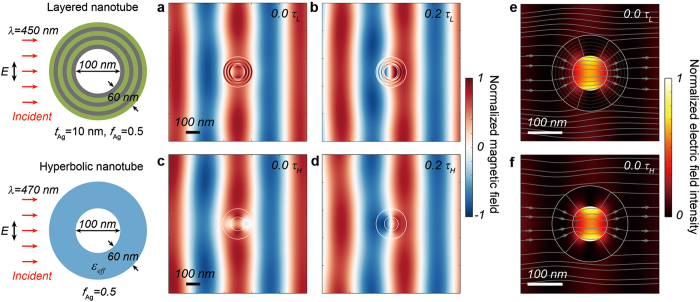
Field distributions in the metal-dielectric layered nanotube and the hyperbolic metamaterial nanotube. (**a**) Time snapshot (0.0*τ*_*L*_) of the normalized magnetic field distribution of the layered nanotube with air core diameter (*D*) of 100 nm, total shell thickness (*T*) of 60 nm, Ag layer thickness (*t*_Ag_) of 10 nm, and TiO_2_ layer thickness (*t*_TiO2_) of 10 nm. The transverse-electric (TE) polarized light with a wavelength of 450 nm was normally incident to the nanotube. (**b**) The same normalized magnetic field distribution as that shown in (**a**), but at a different time (0.2*τ*_*L*_). *τ*_*L*_ is the temporal period of the incident light at the wavelength of 450 nm. (**c**) Time snapshot (0.0*τ*_*H*_) of the normalized magnetic field distribution of the hyperbolic metamaterial nanotube with *D* of 100 nm, *T* of 60 nm, and filling ratio of the Ag layer (*f*_Ag_) of 0.5. The TE-polarized light with a wavelength of 470 nm was normally incident to the nanotube. (**d**) The same normalized magnetic field distribution as that shown in (**c**) but at a different time (0.2*τ*_*H*_). *τ*_*H*_ is the temporal period of the incident light at the wavelength of 470 nm. (**e**) Normalized electric field intensity of the layered nanotube of (**a,b**) at the time of 0.0*τ*_*L*_. The gray lines indicate the time-averaged Poynting vector power flow and the arrows indicate the direction of the power flow. (**f**) Normalized electric field intensity of the hyperbolic nanotube of (**c,d**) at the time of 0.0*τ*_*H*_. All the scale bars are 100 nm.

Finally, we further clarified the validity of our proposed model by investigating and comparing the time evolution of electromagnetic waves at invisible frequencies in both the hyperbolic metamaterial and the metal-dielectric layered nanotubes (Fig. 4). First, the light propagation in the layered nanotube with an air core diameter of 100 nm, Ag and TiO_2_ layer thicknesses of 10 nm, and a total shell thickness of 60 nm was simulated at an invisible wavelength of 450 nm by applying a TE-polarized incident plane wave. Figure 4a,b show the normalized magnetic field distributions near the layered nanotube at two representative time frames of 0.0*τ*_*L*_ and 0.2*τ*_*L*_, respectively, where *τ*_*L*_ is the temporal period of the incident light at the wavelength of 450 nm. For a direct comparison, we also simulated the light propagation in the hyperbolic metamaterial nanotube with the same structural parameters and incident conditions in Fig. 4a,b, but at the different invisible wavelength of 470 nm. In Fig. 4c,d, we also obtained the normalized magnetic field distributions near the hyperbolic metamaterial nanotube at the two representative time frames of 0.0*τ*_*H*_ and 0.2*τ*_*H*_, where *τ*_*H*_ is the temporal period of the incident light at the wavelength of 470 nm. Surprisingly, when compared at the same representative time frames, the normalized magnetic fields evolve in time with the identical temporal phase in both hyperbolic metamaterial and metal-dielectric layered nanotubes. This result reveals that the dynamic behaviors of light scattering and interference of the layered nanotube can be accurately described using the hyperbolic metamaterial nanotube model. In addition, we further investigated the light intensity distributions and power flows in space near the metal-dielectric layered and the hyperbolic metamaterial nanotubes at invisible wavelengths (Fig. 4e,f). The electric field intensity distributions at one of the representative time frames were plotted with the superimposed time-averaged Poynting vector power flows (denoted as gray lines and arrows). As expected, in both nanotubes, the electric field intensities show similar spatial distribution in the nanotube cores and shells as well as surrounding environments. The energy flow through the hyperbolic metamaterial nanotube also reproduced well the flow in the layered nanotube. Moreover, we observed a significant electric field intensity enhancement inside both the hyperbolic metamaterial and metal-dielectric layered nanotubes, which shows the subwavelength confinement of surface plasmons at the interface between the air core and shell structure. This air core has a volume less than ~0.16 (*λ*/2*n*)^2^, where *λ* is the wavelength of the incident light and *n* is the refractive index of the core. Hence, such strong light confinement in the subwavelength region will be particularly advantageous for various nanophotonic applications requiring strong light-matter interaction, high Purcell enhancement of radiative rate as well as spontaneous emission enhancement^28^,^29^. Invisible photodetectors can also be demonstrated by introducing an absorbing material in the air core of the layered nanotube (Supplementary Figure S4).

Based on the light scattering analysis and the dynamics of the field distribution simulation, we found that, for TE-polarized incident light, a metal-dielectric layered nanotube can be accurately described as a single-shell radial-anisotropic hyperbolic metamaterial nanotube with an identical core diameter, total shell thickness, and an appropriate effective permittivity tensor. The equivalence of the layered nanotube to the hyperbolic metamaterial nanotube also holds for the TM-polarized incident light (see Supplementary Figure S5 for details). Thus, the invisible characteristic of the metal-dielectric layered nanotube can be efficiently obtained and tuned using its equivalent hyperbolic nanotube. The hyperbolic dispersion, a consequence of the layered nanotube, was also reproduced well in the equivalent hyperbolic nanotube. From the well-known permittivity of metal and dielectric materials, we can design an invisible layered nanotube by setting the *ε*_*θ*_ of the equivalent hyperbolic metamaterial nanotube to be ENZ at a desired wavelength.

## Discussion

In summary, we theoretically investigated the invisible scattering properties of a metal-dielectric layered nanotube in the visible wavelength regime. By analyzing the light scattering using the Lorentz-Mie method and full-wave simulation, we showed that the invisible wavelength observed in the layered nanotube depends on the filling ratio, rather than the core diameter. In addition, the layered nanotube can be described as a radial-anisotropic hyperbolic metamaterial nanotube. As the invisibility is related to the near-zero effective permittivity in the *θ*-direction of the hyperbolic metamaterial nanotube, an invisible layered nanotube can be easily demonstrated at a desired wavelength. Our efficient way to design and tune the layered nanotube with invisibility will be highly useful for the practical implementation of unique nanophotonic devices such as invisible photodetectors and low-scattering near-field optical microscopes.

## Methods

### Analytic and numerical calculations

To calculate the light scattering of the metal-dielectric layered nanotube and the single-shell hyperbolic metamaterial nanotube, we used the Lorentz-Mie light scattering method (Figs 1b, 2a,b and 3d,e) as described in refs 31,36. The scattering efficiency is defined as *σ*_sca_/(*D* + 2*T*), where *σ*_sca_ is the scattering cross section, *D* is the core diameter, and *T* is the total shell thickness of the nanotube. By using the Lorentz-Mie formalism, we obtained the magnetic and electric field distributions near the metal-dielectric layered nanotube and the hyperbolic metamaterial nanotube (Fig. 4a–d). The Poynting vector power flows were also calculated using the electric and magnetic field results (Fig. 4e–f). In addition, we performed the finite element full-wave simulation for obtaining the magnetic field distribution extending to far-field region, by using COMSOL Multi-physics two-dimensional wave optics module (Fig. 1c,d). The shape of the calculation domain is circular and the radius of the calculation domain is 10*λ*, where *λ* is the wavelength of the incident light. The metal-dielectric layered nanotube was placed at the center of the calculation domain. The perfectly matched layer was applied to eliminate the unwanted reflection from the outer boundary of the calculation domain.

### Permittivities of Ag and TiO_2_

For all simulations and calculations, we adopted the experimental tabulated permittivity functions for Ag and TiO_2_^37^,^38^. The Drude dispersive model was used for Ag: *ε*_Ag_ = 3.691–9.152^2^/((1.24/*λ*)^2^ + i 0.021 (1.24/*λ*)), and the Sellmeier dispersion equation was used for TiO_2_: *ε*_TiO2_ = 5.193 + 0.244/(*λ*^2^ - 0.0803), where *λ* is the wavelength in the unit of micrometer (Supplementary Figure S1).

## Additional Information

**How to cite this article**: Kim, K.-H. *et al.* Invisible Hyperbolic Metamaterial Nanotube at Visible Frequency. *Sci. Rep.* **5**, 16027; doi: 10.1038/srep16027 (2015).

## Supplementary Material

Supplementary Information
