# Supplementary material for: Invisible Hyperbolic Metamaterial Nanotube at Visible Frequency
Source: Sci Rep. 2015 Nov 2;5:16027. doi: 10.1038/srep16027 (PMC4629200; doi:10.1038/srep16027)
Supplement: Supplementary Information [file srep16027-s1.doc]

Supplementary Information for

**Invisible Hyperbolic Metamaterial Nanotube
at Visible Frequency**

*Kyoung-Ho Kim, You-Shin No, Sehwan Chang, Jae-Hyuck Choi, and Hong-Gyu Park^*^*

^*^ Corresponding author. E-mail: [hgpark@korea.ac.kr](mailto:hgpark@korea.ac.kr)

This file includes:

Supplementary Figures S1 – S5

Supplementary References


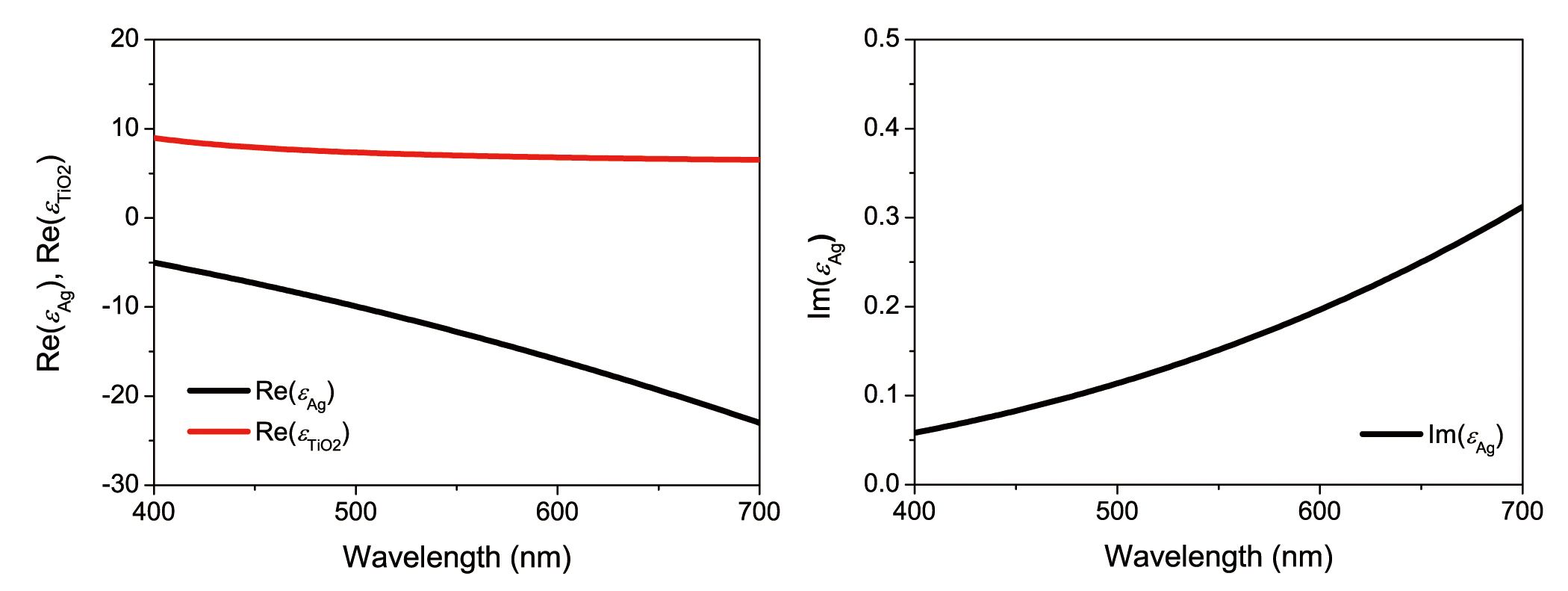


Figure S1. Permittivities of Ag and TiO_2_. Permittivity spectra of Ag and TiO_2_ used in the scattering analysis of the metal-dielectric layered and the hyperbolic metamaterial nanotubes.


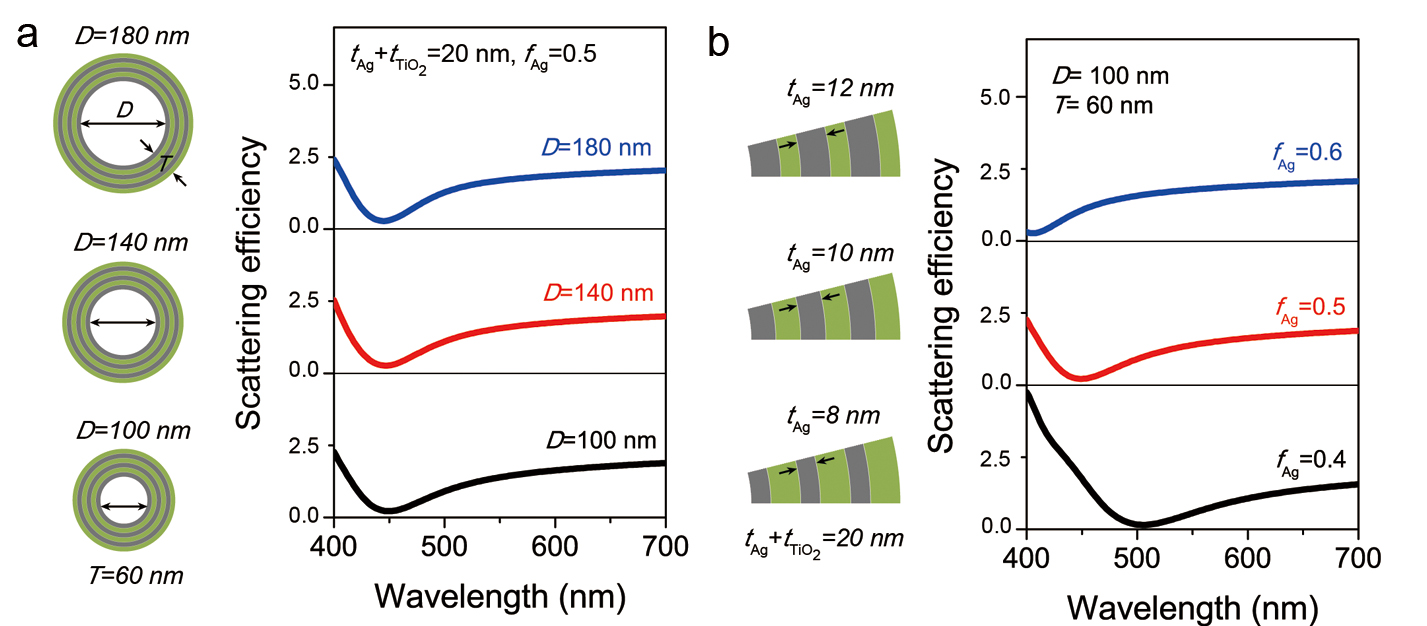


Figure S2. Scattering characteristics of the layered nanotubes with different core diameters and filling ratios for transverse-magnetic (TM) polarized incident light. (a) Scattering efficiency spectra of the layered nanotubes with different core diameter (*D*) for TM-polarized incident light. *D* is changed from 100 nm (bottom) to 180 nm (top), as the thicknesses of the total shell (*T*), Ag layer (*t*_Ag_), and TiO_2_ layer (*t*_TiO2_) are fixed to 60, 10, and 10 nm, respectively. (b) Scattering efficiency spectra of the layered nanotubes with different Ag filling ratio (*f*_Ag_) for TM-polarized incident light. *f*_Ag_ is changed from 0.4 (bottom, *t*_Ag_ = 8 nm) to 0.6 (top, *t*_Ag_ = 12 nm), as *D*, *T*, and the period of the Ag and TiO_2_ layers (*Λ*) are fixed to 100, 60, and 20 nm, respectively.


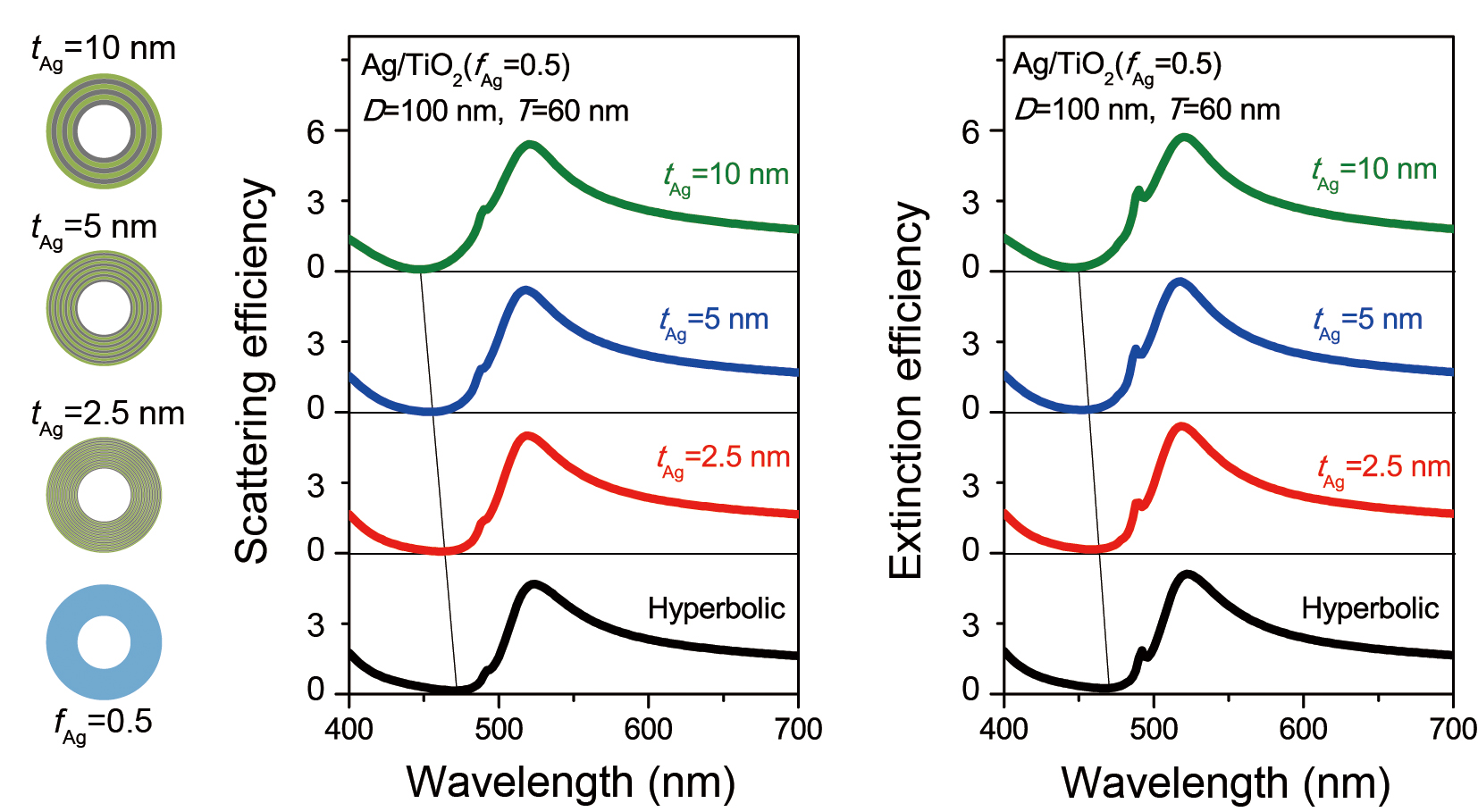


Figure S3. Scattering and extinction efficiency spectra of various layered nanotubes with different Ag/TiO_2_ layer thicknesses for transverse-electric (TE) polarized incident light. The thicknesses of the Ag (*t*_Ag_) and TiO_2_ (*t*_TiO2_) layers are all 2.5 (red), 5 (blue), and 10 nm (green), while the core diameter (*D*), total shell thickness (*T*), and Ag filling ratio *f*_Ag_ are fixed to 100 nm, 60 nm, and 0.5, respectively. For comparison, the scattering and extinction efficiency spectra of a hyperbolic metamaterial nanotube (black) with *f*_Ag_ of 0.5, *D* of 100 nm, and *T* of 60 nm are also shown. As the Ag and TiO_2_ layers are thinner, the scattering and extinction efficiency spectra of the layered nanotube converge to the corresponding scattering and extinction efficiency spectra of the hyperbolic metamaterial nanotube. In addition, the extinction efficiency is slightly larger than the scattering efficiency, particularly at a peak of ~525 nm, due to non-zero optical loss in metal films. However, the difference between the extinction and scattering efficiencies becomes nearly zero and the absorption of the structure is negligible at an invisible wavelength of ~450 nm.


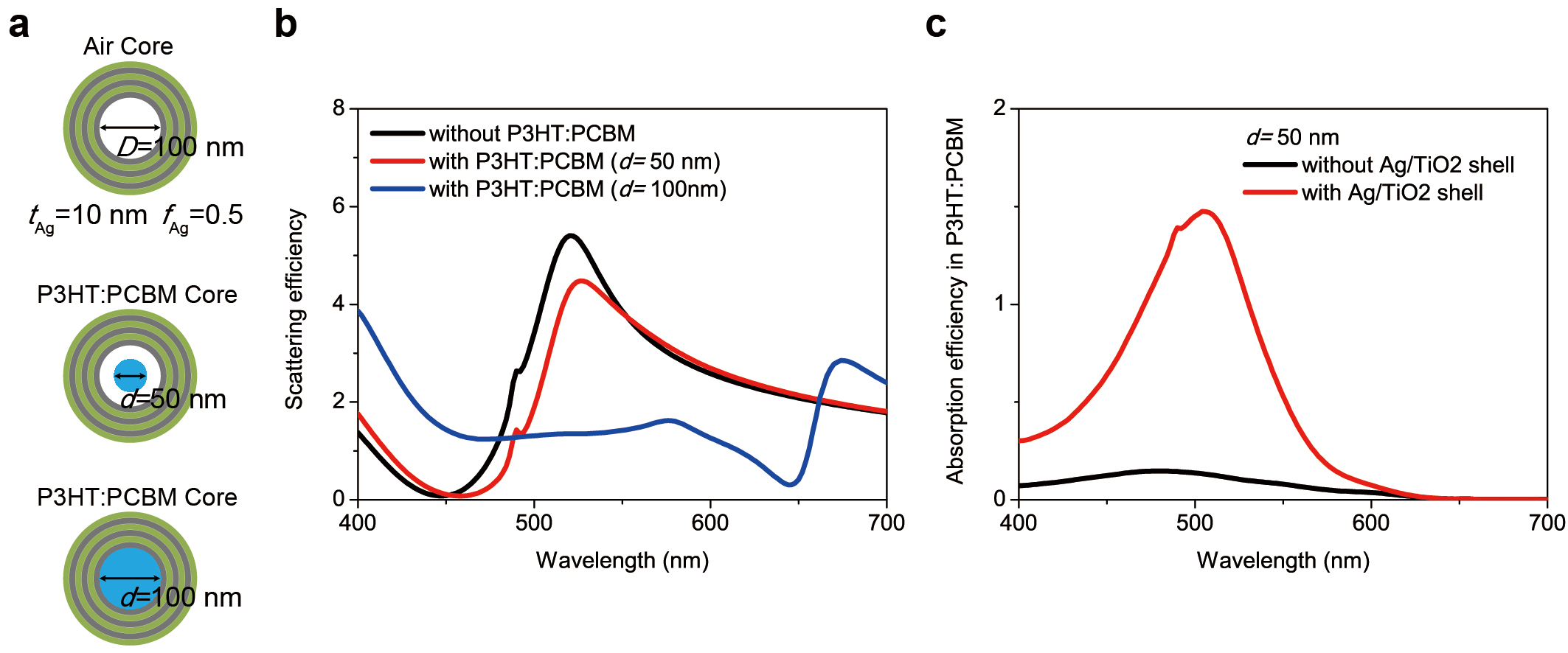
 **Figure S4. Layered nanotubes with absorbing materials in the air cores.** (a) In the layered nanotube, the air core is filled with air (top), P3HT:PCBM nanowire with a diameter of 50 nm (middle), and P3HT:PCBM nanowire with a diameter of 100 nm (bottom). The air core diameter (*D*) and the thicknesses of the total shell (*T*), Ag layer (*t*_Ag_) and TiO_2_ (*t*_TiO2_) are fixed to 100, 60, 10 and 10 nm, respectively. For simulation, we adopted the experimental tabulated permittivity function of P3HT:PCBM which is an absorbing material widely used in organic solar cells.^1,2^ (b) Scattering efficiency spectra of the layered nanotubes without P3HT:PCBM (black) and with 50-nm P3HT:PCBM (red) and 100-nm P3HT:PCBM (blue) in the air cores, for transverse-electric (TE) polarized light. The scattering efficiency spectrum is slightly changed when the 50-nm P3HT:PCBM is introduced in the air core, compared to the original air-core layered nanotube. On the other hand, for the 100-nm P3HT:PCBM in the core, the spectrum changes significantly and the invisible wavelength of ~460 nm disappears. (c) Absorption efficiency spectra of the 50-nm P3HT:PCBM nanowires with (red) and without (black) the air-core layered nanotube of (a). The absorption efficiency of P3HT:PCBM is significantly enhanced by introducing the layered nanotube. In particular, the absorption efficiency is 5.8 times increased at an invisible wavelength of ~460 nm.


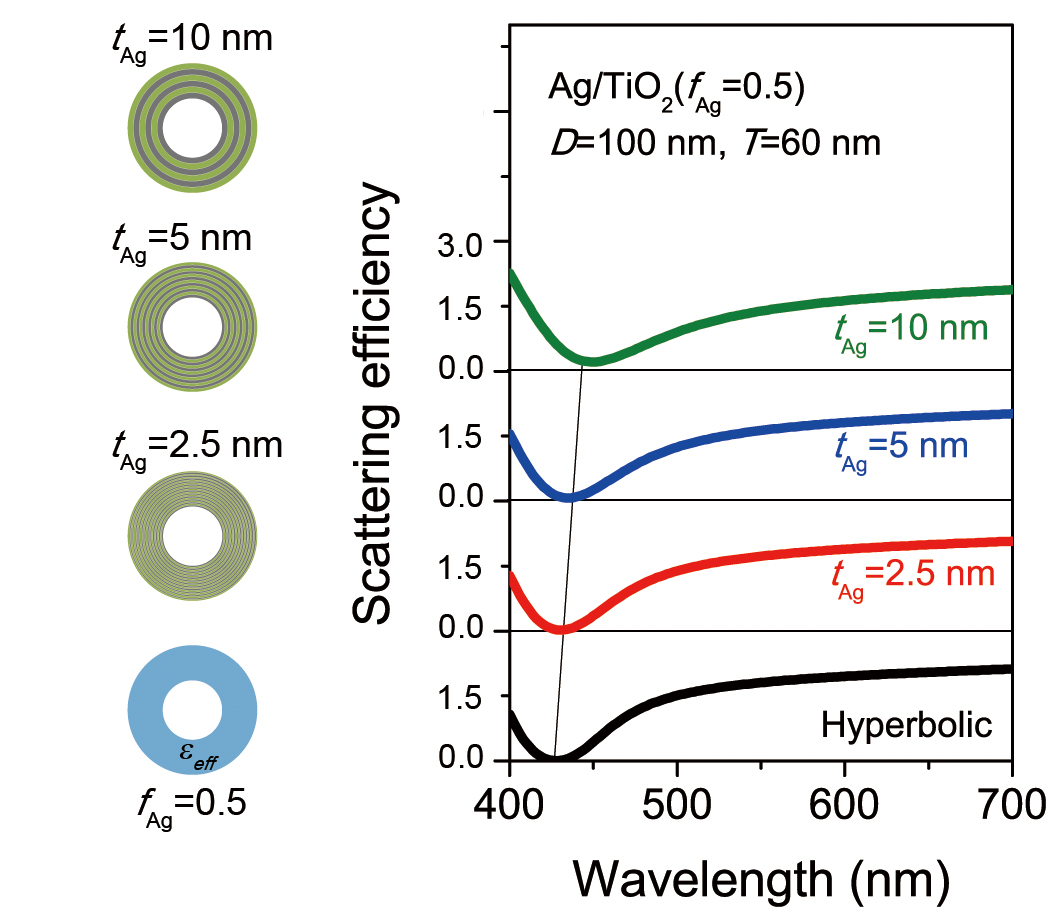


Figure S5. Scattering efficiency spectra of various layered nanotubes with a fixed Ag filling ratio (*f*_Ag_) and different Ag/TiO_2_ thicknesses for transverse-magnetic (TM) polarized incident light. The thicknesses of the Ag (*t*_Ag_) and TiO_2_ (*t*_TiO2_) layers are all 2.5 (red), 5 (blue), and 10 nm (green), as the core diameter (*D*), total shell thickness (*T*), and *f*_Ag_ are fixed to 100 nm, 60 nm, and 0.5, respectively. For comparison, the scattering efficiency spectrum of a hyperbolic nanotube (black) with *D* of 100 nm and *T* of 60 nm is also shown.

**Supplementary References**

1. Li, G., Zhu, R. & Yang, Y. Polymer solar cells. *Nat. Photonics* **6,** 153–161 (2012).

2. Dennler, G. *et al.* Angle dependence of external and internal quantum efficiencies in bulk-heterojunction organic solar cells. *J. Appl. Phys.* **102,** 054516 (2007).
